# Disparities in Insulin Pump Use Among Spanish-Speaking Children With Type 1 Diabetes Compared to Their Non-Hispanic White Peers: Mixed Methods Study

**DOI:** 10.2196/45890

**Published:** 2023-06-09

**Authors:** Lindsey Loomba, Shaila Bonanno, Diana Arellano, Stephanie Crossen, Nicole Glaser

**Affiliations:** 1 Department of Pediatrics University of California, Davis Sacramento, CA United States; 2 Department of Pediatrics University of Washington Seattle, WA United States; 3 UCSF Benioff Children's Hospital San Francisco, CA United States

**Keywords:** disparities, type 1 diabetes, Spanish-speaking, insulin pump, children, diabetes, diabetes mellitus, insulin, glucose monitoring

## Abstract

**Background:**

Disparities in Insulin Pump Use Among Spanish-Speaking Children With Type 1 Diabetes Compared to Their Non-Hispanic White Peers: Mixed Methods Study

**Objective:**

We aimed to investigate the use of insulin pumps and continuous glucose monitoring (CGM) devices among Spanish-language–preferring children in our clinic population and to identify specific barriers to technology use.

**Methods:**

First, we assessed rates and patterns of diabetes technology use (eg, insulin pumps and CGM devices) in a sample of 76 children (38 Spanish-language preferring and 38 non-Hispanic White). We compared rates of technology use, average length of time between diabetes diagnosis and initiation of insulin pump or CGM device, and rates of discontinuation of these devices between the Spanish-language–preferring and non-Hispanic White children. Second, to understand specific barriers to technology use, we compared responses to a questionnaire assessing decision-making about insulin pumps.

**Results:**

Spanish-language–preferring patients had lower rates of insulin pump use, even after controlling for age, gender, age at diagnosis, and type of health insurance. Spanish-language–preferring participants were more likely to report concerns over learning to use an insulin pump and were more likely to discontinue using an insulin pump after starting one.

**Conclusions:**

These data confirm demographic disparities in insulin pump use among children with T1D and provide new insights about insulin pump discontinuation among Spanish-language–preferring children. Our findings suggest a need for improved patient education about insulin pump technology in general and improved support for Spanish-language–preferring families with T1D after initiation of pump therapy.

## Introduction

In children with type 1 diabetes (T1D), insulin pumps and continuous glucose monitoring (CGM) devices can improve glycemic control, decrease rates of severe hypoglycemia and diabetic ketoacidosis, and reduce risk of microvascular complications [1,2]. Prior work has reported racial disparities in both diabetes outcomes and rates of technology use among Hispanic children compared to non-Hispanic White children [3-8]. Further, differences in attitudes about diabetes technology between ethnic groups have also been identified [9]. The specific effects of a language barrier on diabetes technology uptake are not fully understood, and we hypothesized that children from Hispanic families with a language barrier (ie, those whose families identify Spanish as their primary language) would be less likely than their White counterparts to use diabetes technologies and might have differing barriers to technology use. Our study was thus designed to determine rates and patterns of insulin pump and CGM device use among Spanish-language–preferring Hispanic children with T1D receiving care at an academic medical center and to further identify specific barriers to technology use in these children. A recent study [9] has shown differences in technology use between Spanish-speaking and English-speaking Latinx families, with significant differences found in technology use and attitudes about diabetes technology between the 2 language groups. Our study, therefore, builds upon what is currently known about differences in care outcomes among children with T1D by examining specific experiences and potential barriers to diabetes technology use among Spanish-language–preferring patients with T1D compared to their White peers.

## Methods

### Ethics Approval

Study approval was provided by the UC Davis Institutional Review Board (assessing rates and patterns: 1458281-2; assessing barriers: 1460830-1), and consent requirements were waived for this portion of the study.

### Procedure

First, to assess differences in rates and patterns of diabetes technology use, we evaluated medical records for 76 children (38 Spanish-language preferring and 38 non-Hispanic White) with T1D who received their routine diabetes care at our academic children’s hospital. Spanish-language–preferring children were eligible for medical record review if they were aged 0-18 years, had a prior diagnosis of T1D, had received care in our diabetes clinic in the prior 12 months, and identified Spanish as their family’s primary language (indicated by demographic data in their electronic health records and verified by study personnel). Children were excluded if they lived with a household member also diagnosed with T1D (n=1) and had a severe allergy to adhesive material (n=0) or if there were significant learning disabilities in the child or either parent (n=1), due to the likelihood of these factors influencing diabetes technology uptake. All of the Spanish-language–preferring children seen in our practice who met the inclusion and exclusion criteria were included in the study. The same inclusion and exclusion criteria were used for the non-Hispanic White participants, except for primary language (which was required to be English). From among the pool of eligible non-Hispanic White patients, participants were selected via random matching to the Spanish-language–preferring participants based on current age and diabetes duration using a computer algorithm. Families were not compensated for participation in this portion of the study, which only involved medical record review.

From each participant’s electronic health record, we recorded the date of T1D diagnosis, age at diabetes diagnosis, diabetes duration, gender, ethnicity, and type of health insurance (public vs private). Data on technology use were collected, including if the child had ever used an insulin pump or CGM device, if the child was currently using an insulin pump or CGM device, and the dates the insulin pump or CGM device were used. Participant selection and data abstraction were both performed in 2019.

We then compared the rates of technology use (eg, insulin pump and CGM device), the average length of time between diabetes diagnosis and initiation of technology use, and the rates of device discontinuation between the Spanish-language–preferring and non-Hispanic White groups. We initially compared data for Spanish-language–preferring participants to the data of non-Hispanic White participants using 2-tailed student *t* test for continuous variables and chi-square test for categorical variables. We then used logistic regression analyses to determine whether differences in technology use persisted after adjusting for the effects of other covariates, such as sex and type of health insurance.

Second, to better understand specific barriers to technology use, all Spanish-language–preferring participants and their families identified from the previous cohort were asked to complete a written questionnaire detailing their decision-making surrounding technology use. The questionnaire contained items pertaining to both pump and CGM use; however, as Spanish language preference was not predictive of CGM use in the first portion of the study, we did not conduct further analyses on survey responses related to CGM use. Questions and response options included in the questionnaire can be accessed in Table S1 in Multimedia Appendix 1. Translation of the questionnaire from English to Spanish was provided by the institution’s interpreting and translation services department. Participants for this portion of the study were enrolled by the study personnel during outpatient diabetes visits over a 6-month period in 2019-2020. For this portion of the study, participants received a US $20 gift card as compensation.

To gather comparable perspectives about insulin pump nonuse by non-Hispanic White patients, we identified a cohort of non-Hispanic White patients who met our initial inclusion criteria but were not using insulin pumps. These participants were again matched to our Spanish-language–preferring cohort based on age and diabetes duration. Non-Hispanic White control participants were enrolled at the time of a regularly scheduled clinic visit, similar to our Spanish-language–preferring participants, and completed the same written questionnaire about diabetes technology decision-making during the same study period. All non-Hispanic White control participants completed the questionnaire in English. The non-Hispanic White survey group was selected for pump nonuse (to capture an adequate sample of pump nonusers from among an ethnic cohort with a majority using the pump); therefore, the non-Hispanic White sample was larger than the Spanish-language–preferring sample for this part of the analysis. We did not select for insulin pump never-use to allow for analysis of rates of pump discontinuation. Questionnaire responses in the Spanish-language–preferring and non-Hispanic White groups were compared using Fisher exact test.

## Results

### Participant Selection

A total of 43 children with T1D aged 0-18 years with Hispanic ethnicity and Spanish-language preference, who were listed in the electronic medical record, were initially identified and had their primary language confirmed by the study personnel. Of these, 2 failed to meet all inclusion criteria, and 3 lacked information in their medical records necessary for accurate data retrieval. Thus, 38 Spanish-language–preferring children were included for analyses assessing rates of technology use. In addition, 583 non-Hispanic White children were identified as potential controls, and 38 of them were matched to the Spanish-language–preferring participants via an Microsoft Excel-based computerized formula using date of birth and date of diabetes diagnosis.

### Rates of Technology Use

Demographic characteristics of the study groups are shown in Table 1.

In univariate analyses, Spanish-language–preferring participants were less likely to use both insulin pumps (13/38, 34% vs 24/38, 63%; *P*=.01) and CGM devices (19/38, 50% vs 30/38, 79%; *P*=.01). Among families using pumps, the Spanish-language–preferring participants started use at approximately the same time after diagnosis as the non-Hispanic White participants (26.6 months vs 25.7 months; *P*=.91). CGM device use began later in Spanish-language–preferring participants (42.3 months vs 24.4 months; *P*=.07), but the difference was just short of statistical significance. Of note, there were significantly more patients with public insurance in the Spanish-language–preferring group than in the non-Hispanic White group (35/38, 92% vs 13/38, 34%). Overall, those with public insurance were significantly less likely to use a CGM device (*P*=.01), but there was no difference in insulin pump use by health insurance type (*P*=.11).

In multivariable analyses, ethnicity or language group and diabetes duration continued to be significant predictors of insulin pump use, after adjusting for age, gender, age at diagnosis, and type of insurance (Table 2).

**Table 1:** Demographic and clinical characteristics of the study participants (N=76)

Characteristics	Spanish-language preferring (n=38)	Non-Hispanic White (n=38)	*P* value for comparison of the groups
Age (years), mean (SD)	11.9 (3.3)	11.8 (3.4)	N/A^a^
Age at diagnosis (years), mean (SD)	7.1 (3.9)	7.1 (3.9)	N/A
Diabetes duration (years), mean (SD)	4.8 (3.8)	4.7 (3.8)	N/A
Sex (male), n (%)	12 (32)	19 (50)	.10
Public insurance, n (%)	35 (92)	13 (34)	<.01
Current pump use, n (%)	13 (34)	24 (63)	.01
Current CGM^b^ device use, n (%)	19 (50)	30 (79)	.01

^a^N/A: not applicable.

^b^CGM: continuous glucose monitoring.

**Table 2:** Predictors of diabetes technology use (multivariable analyses).

Variable		Coefficient (95% CI)	*P* value
**Insulin pump use**			
	Spanish-language preference	–1.38 (–2.62 to –0.13)	.03
	Male sex	–0.03 (–1.11 to 1.05)	.96
	Public insurance	0.06 (–1.27 to 1.39)	.93
	Diabetes duration	0.02 (0.004 to 0.03)	.01
	Age	–0.06 (–0.21 to 0.09)	.45
**CGM^a^ device use**			
	Spanish-language preference	–0.95 (–2.22 to 0.32)	.14
	Male sex	–0.54 (–1.68 to 0.59)	.35
	Public insurance	–0.91 (–2.38 to –0.57)	.23
	Diabetes duration	–0.01 (–0.02 to 0.004)	.20
	Age	–0.05 (–0.22 to 0.13)	.61

^a^CGM: continuous glucose monitoring.

In regard to CGM device use, ethnicity was no longer predictive of device use after adjusting for age, gender, age at diagnosis, and type of insurance. Analyses of barriers to technology use therefore focused on participants who were not using insulin pumps, and we did not conduct further analyses pertaining to CGM use.

### Barriers to Technology Use

Of the 38 Spanish-language–preferring families identified through medical record review, 30 were seen in clinic during the 6-month study period and completed the questionnaire assessing barriers to technology use. These 30 participants were then matched to a group of 30 non-Hispanic White pump nonusers based on age and diabetes duration (Table 3).

Of the Spanish-language–preferring participants, 19 were current pump nonusers, and 13 had never previously used an insulin pump. To avoid having prior pump users reporting on decision-making about pump use, only the participants who had never used a pump were included in these analyses. Although the non-Hispanic White control participants were selected based on current insulin pump nonuse (not insulin pump never-use), none had ever previously used a pump.

In response to questions regarding pump use, Spanish-language–preferring patients were far more likely to report previously using an insulin pump but discontinuing it due to dislike of the technology (6/19, 32% vs 0/30, 0%; *P*=.002; Table 4). There were not differences seen between the 2 groups with regard to primary reason(s) for pump nonuse, such as lack of perceived need or fear of error (Table 5).

In questions assessing familiarity with pumps among insulin pump never-users, no difference was seen between the Spanish-speaking (n=13) and non-Hispanic White (n=30) groups in terms of having ever seen someone use an insulin pump (7/13, 54% vs 25/30, 83%; *P*=.06) or in whether they had discussed pump use with health care providers (11/13, 85% vs 29/30, 97%; *P*=.21).

In the question assessing impressions and major concerns about pump use, Spanish-language–preferring participants were less likely to report confidence in learning to use the device (median questionnaire score 3 vs 5; *P*=.001) and more likely to cite concern over cost (median questionnaire score 4 vs 2; *P*=.05) compared to non-Hispanic White participants.

**Table 3:** Characteristics of pump nonusers reporting on technology barriers.

	Spanish-language preferring (n=19)^a^	Non-Hispanic White (n=30)	*P* value for comparison of the groups
Age (years), mean (SD)	12.9 (2.7)	12.9 (4.1)	.98
Age at diagnosis (years), mean (SD)	8.8 (3.9)	8.8 (3.9)	.98
Diabetes duration (years), mean (SD)	4.1 (3.4)	4.1 (3.3)	.97
Current CGM^b^ device use, n (%)	9 (47)	19 (63)	.27

^a^n=19 due to inclusion of pump nonusers only.

^b^CGM: continuous glucose monitoring.

**Table 4:** Pump discontinuation rates among insulin pump nonusers.

Rates of pump discontinuation	Spanish-language preferring (n=19)	Non-Hispanic White (n=30)	*P* value for comparison of the groups
History of prior pump use (cited “previously tried/didn’t like” on survey), n (%)	6 (32)	0 (0)	.002

**Table 5:** Reasons cited for insulin pump nonuse among insulin pump never-users.

Reason	Spanish-language preferring (n=13^a^), n (%)	Non-Hispanic White (n=30), n (%)	*P* value for comparison of the groups
No perceived need	4 (30)	11 (37)	>.99
Difficult to understand	4 (30)	6 (20)	.46
Does not want something attached	5 (39)	15 (50)	.53
Cost	1 (8)	1 (3)	.52
Fear of error	2 (15)	10 (33)	.29
Did not qualify for use	1 (8)	1 (3)	.52

^a^n=13 due to inclusion of participants who had never used an insulin pump. Totals do not equal 100% as study participants could select all answers that applied.

## Discussion

### Principal Findings

Recent literature has highlighted racial and ethnic disparities in both glycemic outcomes and diabetes technology use among children with T1D [3-10]. Common barriers to pump therapy adoption in pediatric patients include concerns about having a device attached to the body, therapeutic effectiveness compared to insulin injection regimens, and cost burden [11-13]. Additional barriers faced by historically marginalized racial and ethnic groups can include difficulties with access to care, provider bias, and socioeconomic disparities [14-16]. The specific effect of a language barrier on diabetes technology use is uncertain in the existing pediatric literature, and preliminary data [9] suggest technology use and attitudes may vary among Spanish-speaking versus English-speaking Hispanic patients and families. Our study was designed to explore specific experiences and potential barriers to diabetes technology use among Spanish-language–preferring patients with T1D.

Our results are consistent with the existing literature in that we found lower rates of insulin pump use in Spanish-language–preferring children compared to non-Hispanic White controls. This finding held true even after adjusting for age, sex, diabetes duration, and type of insurance. However, we did not find that ethnicity was a significant predictor of CGM device use after adjustment for health insurance type, which was strongly associated with CGM device use in our study population. Differences in access to CGM device based on health insurance may therefore have obscured differences related to ethnicity within our cohort. Of note, our clinic protocol for CGM initiation was relatively straightforward compared to the protocol for insulin pump initiation at the time of this study. For pump initiation, our patients were required to attend a pre–pump use class (during which they learned about insulin pump therapy and various device options) and complete a pre–pump use checklist, which included a skills assessment on various aspects of diabetes management. For CGM initiation, our patients indicated interest to their diabetes provider and received basic information on the device from a registered nurse/certified diabetes care and education specialist. It is possible that the process for pump initiation presented additional barriers to our Spanish-language–preferring patients and that this contributed to the differences seen between pump and CGM use in our clinic.

In other settings, provider bias has been widely identified as a contributing factor to racial and ethnic disparities in health outcomes [17,18]. In our study, reported frequencies of discussion about diabetes technologies with care providers were similar between our Spanish-speaking and non-Hispanic White groups, suggesting that bias in clinician’s decisions about introducing diabetes technology may not have played a major role in the differences we observed. However, our questionnaire did not assess the content of these clinician discussions, which may have influenced whether Spanish-language–preferring participants felt adequately educated and encouraged about technology use. The fact that our Spanish-language–preferring families felt less confident that they could learn to use a pump suggests that additional education from their health care teams may be needed to prepare them for successful technology use.

In sum, our findings suggest that further work is needed to better understand how to best support diabetes technology use among the Spanish-language–preferring community. Improved Spanish language teaching materials and in-person Spanish instructions are likely needed as well as increased contact with peer groups using diabetes technology, to reduce the observed disparities in insulin pump use between Spanish-language–preferring patients and their non-Hispanic White peers. In addition, our novel finding that Spanish-language–preferring children are more likely to discontinue insulin pump use after starting it highlights the need for improved support after initiation of pump therapy. Shared medical appointments that include group education have been associated with increased technology adoption among Spanish-language–preferring children and adolescents [19], but additional studies are needed to determine how to best maintain insulin pump and CGM device use in these patients and families.

### Strengths and Limitations

A strength of our study was the relatively high study completion rates for eligible participants, minimizing issues with sampling bias. However, the study also has several limitations. First, the study sample was small due to the single center analysis, and generalizability is limited by possible variations in clinical practice and patterns of insurance coverage. In addition, we did not collect information on socioeconomic status beyond insurance type, and this could be an important variable to consider in future studies, as Spanish-speaking populations may vary culturally and socioeconomically between locations. A larger sample size or alternate study format (eg, focus groups) might allow for additional analyses not conducted in our sample, such as a detailed investigation of reasons why children discontinued insulin pump use. A larger sample size would also be necessary to compare decision-making among Hispanic Spanish-language–preferring families versus Hispanic English-language–preferring families [9].

Additionally, it is possible that our questionnaire failed to capture some barriers to technology use in this patient population. We employed an experienced multidisciplinary diabetes team (including members with Spanish-language fluency and extensive experience working with the Spanish-speaking population) to collaborate in questionnaire design, but the questionnaire was not previously studied or validated for this clinical question and target population, so this remains a limitation. Finally, the study was conducted before the COVID-19 pandemic, and diabetes technology use has changed in several ways since the data were collected. In particular, CGM device use has increased substantially in our patient population due to expanded insurance coverage, particularly among those with public insurance. In addition, several new integrated pump-CGM systems providing automated insulin delivery have been released since our study concluded, and questions of access, use, and comfort with these new systems among Spanish-language–preferring children is an important area for future inquiry.

### Conclusions

This study confirms that Spanish-language preference is associated with lower rates of insulin pump use in children with T1D, even after controlling for age, gender, age at diagnosis, and type of insurance. In addition, our analysis suggests that Spanish-language–preferring families experience higher rates of insulin pump discontinuation than their English-speaking non-Hispanic White counterparts. This finding has not previously been reported in the pediatric T1D literature. Finally, our study demonstrates that Spanish-language–preferring families are more likely than non-Hispanic White controls to report concerns over learning to use insulin pumps, highlighting the need for improved Spanish-language instructions about insulin pumps and increased support for Spanish-language–preferring families after pump technology has been adopted.

## Appendix

Multimedia Appendix 1 Questions and response options included in the questionnaire.

## Abbreviations

CGM: continuous glucose monitoring
T1D: type 1 diabetes
