# Supplementary material for: Disparities in Insulin Pump Use Among Spanish-Speaking Children With Type 1 Diabetes Compared to Their Non-Hispanic White Peers: Mixed Methods Study
Source: JMIR Diabetes. 2023 Jun 9;8:e45890. doi: 10.2196/45890 (PMC10334715; doi:10.2196/45890)
Supplement: Multimedia Appendix 1 [file diabetes_v8i1e45890_app1.docx]

**Supplementary Table S1. Diabetes Technology Use Questionnaire.**

| Primary reason/s for not using an insulin pump |
| --- |
| Why do you not use an insulin pump? Select all that apply.   - Tried it in the past and didn’t like it - Don’t want something attached to my body all the time - Too expensive - Am not testing blood sugar often enough to be approved for one - Worried that pump will be difficult to understand and use - Worried that the pump may make a mistake and cause high or low blood sugar - Don’t see need for it now |
| Familiarity with insulin pumps |
| Have you ever seen someone use an insulin pump?   - Yes - No - If yes, who? _______________________ |
| Has your diabetes doctor or nurse ever talked to you about using an insulin pump?   - Yes - No |
| Current questions about using an insulin pump |
| What types of questions did you have after seeing a pump used? Select all that apply.   - Questions about how the pump works - Questions about why a pump might be better than shots - Questions about keeping it attached all the time - Questions about whether or not child would like it - Questions about what to do if it breaks - Questions about how much it costs |
| Impressions and concerns about insulin pump use |
| Please rate the following on a scale of 1 to 5:   - I feel confident I could learn to use an insulin pump correctly   (not confident at all) 1 2 3 4 5 (very confident)   - How much would it bother you to have something attached to your body?   (not bothered at all) 1 2 3 4 5 (extremely bothered)   - Are you worried that the pump would interfere with daily activities?   (not worried at all) 1 2 3 4 5 (extremely worried)   - How concerned are you about the cost of the pump?   (not concerned at all) 1 2 3 4 5 (extremely concerned)   - How much do you think a pump would improve your blood glucose control?   (no improvement at all) 1 2 3 4 5 (major improvement)   - How useful would the following pump features be?  1. Getting rid of shots   (not useful at all) 1 2 3 4 5 (extremely useful)   1. Not having to carry around so many supplies   (not useful at all) 1 2 3 4 5 (extremely useful)   1. More precise insulin dosing   (not useful at all) 1 2 3 4 5 (extremely useful)   1. Pump will do the math and calculate pre-meal insulin doses   (not useful at all) 1 2 3 4 5 (extremely useful) |

Legend: Diabetes technology use questions completed by Spanish-speaking and non-Hispanic white participants and/or their family members (non-pump users only). Questions assessing CGM device use were structured similarly (not shown).
